# Gastrocnemius Muscle Structural and Functional Changes Associated with Domestication in the Turkey

**DOI:** 10.3390/ani11071850

**Published:** 2021-06-22

**Authors:** Kristin K. Stover, David A. Sleboda, Elizabeth L. Brainerd, Thomas J. Roberts

**Affiliations:** 1 The Department of Ecology and Evolutionary Biology, Brown University, Providence, RI 02912, USA; david.sleboda@mcgill.ca (D.A.S.); elizabeth_brainerd@brown.edu (E.L.B.); thomas_roberts@brown.edu (T.J.R.); 2 Department of Biomedical Science, West Virginia School of Osteopathic Medicine, Lewisburg, WV 24901, USA

**Keywords:** domestic turkey, wild turkey, intramuscular connective tissue, isometric force, muscle fiber, scaling, muscle hyperplasia, collagen

## Abstract

**Simple Summary:**

Domestic turkeys have been bred to reach a body mass of up to three times that of wild turkeys. Most of this increase is from larger muscles, but it is unclear exactly how the components of each muscle have been altered and what that may mean for muscle function. In this study, we looked for potential differences between wild and domestic turkeys in (1) the size of the individual muscle fibers and (2) the collagenous skeleton that supports those fibers in the lateral gastrocnemius muscle, an ankle extensor important for walking. We also measured the force this muscle could produce. The domestic turkey muscle had a greater number of smaller muscle fibers than the wild turkey. The amount of collagen in the domestic turkey muscle was also lower than wild turkeys, likely contributing to meat tenderness. While the domestic turkey lateral gastrocnemius muscles could produce the same amount of force per a given area of muscle, they could only produce half as much force per unit body mass. Selection for increased muscle mass has altered the structure of the lateral gastrocnemius muscle; however, overall body mass likely contributes more to hind limb functional differences observed in the domestic turkey.

**Abstract:**

Selection for increased muscle mass in domestic turkeys has resulted in muscles twice the size of those found in wild turkeys. This study characterizes muscle structural changes as well as functional differences in muscle performance associated with selection for increased muscle mass. We compared peak isometric force production, whole muscle and individual fiber cross-sectional area (CSA), connective tissue collagen concentration and structure of the lateral gastrocnemius (LG) muscle in wild and adult domestic turkeys. We also explored changes with age between juvenile and adult domestic turkeys. We found that the domestic turkey’s LG muscle can produce the same force per cross-sectional area as a wild turkey; however, due to scaling, domestic adults produce less force per unit body mass. Domestic turkey muscle fibers were slightly smaller in CSA (3802 ± 2223 μm^2^) than those of the wild turkey (4014 ± 1831 μm^2^, *p* = 0.013), indicating that the absolutely larger domestic turkey muscles are a result of an increased number of smaller fibers. Collagen concentration in domestic turkey muscle (4.19 ± 1.58 μg hydroxyproline/mg muscle) was significantly lower than in the wild turkeys (6.23 ± 0.63 μg/mg, *p* = 0.0275), with visible differences in endomysium texture, observed via scanning electron microscopy. Selection for increased muscle mass has altered the structure of the LG muscle; however, scaling likely contributes more to hind limb functional differences observed in the domestic turkey.

## 1. Introduction

The domestic turkey has undergone major morphological changes due to artificial selection for high growth rates and increased muscle mass, as well as advances in nutrition that have led to an increase in body mass [1]. This has resulted in turkeys that reach a body mass three times that of their wild counterparts, with most individual muscles reaching a mass at least twice that of wild turkeys [2]. Poultry researchers have documented many variations in meat quality associated with domestication and processing, including alterations to pH after slaughter, presence of PSE (pale, soft, exudative) meat, muscle glycogen levels and tenderness [3,4,5]. In addition to its influence on meat quality, we hypothesize that domestication has influenced structural and functional properties of muscle relevant to its role as a force-producing motor organ. Such changes remain largely unstudied, despite their relevance to the locomotor abilities and overall health of domestic birds. 

Domestic turkeys experience locomotor issues due to genetics, growth rate and body conformation, among other factors [6,7,8]. Steps have been taken to preserve domestic turkey walking ability, including selecting turkeys for breeding with the best gait score, based on an observer’s categorical judgment of a turkey’s walk [9,10]. An investigation of the kinematic and kinetic gait differences between wild and domestic turkeys revealed that domestic turkeys walk more slowly, maintain relatively lower ground reaction forces and have a distinct gait from wild turkeys—changes which all likely contribute to increased stability [11,12]. The reduction in speed and peak ground reaction forces could be partially functional limitations within the hind limb muscles. Selection for muscle size, rather than function and performance, may be compromising the force-producing capabilities of these muscles.

Skeletal muscle is made up of both contractile and connective tissue elements, and changes to either component can affect muscle function [13,14,15]. Myopathies, PSE meat content and variations in white striping are all conditions associated with domestication [16,17,18,19]. Each of these conditions represents changes to the contractile and connective tissue elements of muscle. On a basic level, it remains unclear how increases in muscle mass have been structurally achieved in the domestic turkey. The increase in muscle mass could be due to muscle fiber hypertrophy or an increase in the number of muscle fibers, i.e., hyperplasia. Muscle shape change, either in architecture or overall shape, that occurs with an increase in a muscle’s mass is also important, as they affect the muscle physiological cross-sectional area (PCSA), which is proportional to force production. Alterations in intramuscular connective tissue associated with myopathies have also been detailed in the turkey [19], but baseline differences between wild and domestic turkeys have not yet been described.

Skeletal muscle structural changes are important to understand not only for animal health and welfare, but also for meat quality purposes. The proportions of contractile and connective tissue components affect meat texture. For example, increased connective tissue makes cooked meat tougher and less tender [20]. Understanding how turkey muscle has increased in size, from wild to domestic turkeys, will help to enlighten further breeding and rearing strategies. Intramuscular connective tissue can be manipulated, e.g., with exercise [21]; however, this must be balanced with functional consequences in the muscle and animal. Increased intramuscular collagen has been shown to stiffen muscle and reduce peak isometric force [13]; on the other hand, decreased connective tissue may reduce resistance to muscle bulging, altering gearing and direction of forces in a pennate muscle [22].

In this study, we investigate structural and functional differences in the lateral gastrocnemius muscles of domestic and wild turkeys. We also explore how domestic turkey muscle changes structurally and mechanically during ontogeny to better understand how increased muscle mass may affect locomotor performance at different life stages. We use adult wild turkey muscle as a control group for optimal mechanical performance in this species. The lateral gastrocnemius functions as an ankle extensor crucial to normal locomotion in turkeys, and extensive work characterizing this muscle’s mechanical properties and in vivo performance in wild turkeys is available [23,24,25,26]. Here, we compare peak isometric force produced by the lateral gastrocnemius muscle of each turkey strain, changes in muscle fiber cross-sectional area contributing to muscle mass increase, differences in collagen concentration within the intramuscular connective tissue and observed distributions of collagen among structural levels via scanning electron microscopy (SEM), all of which are features that may be associated with selection for increased muscle mass in the domestic turkey. 

## 2. Materials and Methods 

### 2.1. Animals

Broad breasted white turkeys, *Meleagris gallopavo*, were obtained from local farms between 2013 and 2016. Turkeys were either housed and raised in the Animal Care facilities at Brown University or euthanized immediately upon arrival. Six juvenile domestic turkeys raised on pasture were obtained between 8–12 weeks in age from a local farm. Collagen samples, microscopy samples and force measurements were taken upon arrival to the laboratory. Adult turkeys were all over 18 weeks old when experiments were performed. Ages of data collection for muscle collagen concentrations and SEM image analysis can be found in the supplementary data table. Adult domestic turkeys were from 2 cohorts (*n* = 6 in 2013 and *n* = 4 in 2016). Muscle force data was collected from all ten, while collagen samples were only taken from the four individuals in 2016. 

Wild turkeys were also kept in the animal care facilities, with some obtained 3 days post-hatch and others obtained as adults post-breeding. Muscle samples for collagen measurements and imaging were taken from four adult wild turkeys from the 2015 cohort raised in our facilities. Force data were collected from 20 total wild turkeys in conjunction with other ongoing studies within the lab. For ten of the twenty wild individuals, force measurements were obtained between the ages of 18 weeks and just over one year old. For the remaining ten, force measurements were obtained during previous data collections from birds >3 years old. Please note, wild turkeys are much longer lived than domestic turkeys, so wild turkeys could be sampled over a wider age range.

All turkeys were maintained on an ad libitum water and 28% protein commercial poultry diet for the first 8 weeks and then transitioned to 21% protein poultry feed. Birds raised on pasture likely also ate insects in their environment. Oyster shell grit was provided for calcium, and all birds received mealworm treats during concurrent locomotion studies. The flock lived in an open pen system approximately 300 ft^2^ in area, with a minimum of 15 ft^2^ per individual. The floor was concrete with ample wood shavings, environmental enrichment devices and structures for roosting. The Brown University Institutional Care and Use Committee approved all animal use, IACUC no. 1602000189, and complied with state and federal legislation and regulation. Individual bird information can be found in the Supplementary Information, Table S1.

### 2.2. In Situ Experiment

The surgical and experimental procedure for measuring peak isometric muscle force closely followed previous methods [25,27,28]. Turkeys were deeply anesthetized and the bony tendon of the M. gastrocnemius pars lateralis (LG) was attached to a linear actuator (Kollmorgen EC3-AKM42G, Radford, VA, USA) and tension load cell (Omega Engineering Inc. LC703-500, Norwalk, CT, USA). The tibial branch of the sciatic nerve was stimulated to cause muscle contraction at various muscle fiber lengths, prescribed by the linear actuator, generating a length-tension curve for each muscle. Two sonomicrometry crystals (Sonometrics Inc., London, ON, Canada) were implanted in the muscle along a proximal muscle fascicle to measure fiber length changes during contractions. The tetanic length-tension curve yielded maximum force (P_0_) for each individual turkey. Animals were euthanized at the completion of measurements.

The physiological cross-sectional area of the muscle was calculated with the equation:

(1) $$PMA=m\cdot cos\theta /\rho \cdot L_{f},$$

where *m* is the mass of the muscle in g, *θ* is the pennation angle in degrees, *ρ* is density of 1.06 g/cm^3^ [29] and *L_f_* is muscle fascicle length in cm. Wild turkey data were taken from previous wild turkey experiments in the lab, including [30], and from 3 wild turkeys specifically allocated for this study and method validation.

**Figure 1:**
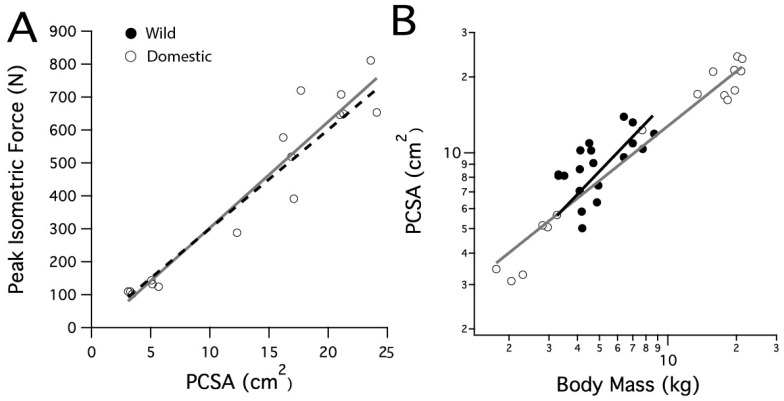
Scaling relationships for the lateral gastrocnemius muscle’s physiological cross-sectional area and peak isometric force. (**A**) The peak isometric force increases with PCSA in the domestic turkey. The black dashed line is calculated from the predicted peak isometric force using 30 N cm^−2^. (**B**) PCSA scales similarly with body mass in the wild and domestic turkeys.

Vertebrate skeletal muscle force is reasonably uniform in proportion to fiber cross-sectional area, ranging from 150 to 300 kPa, with an average of 200 kPa [31]. A prediction for the peak isometric force based on the domestic turkeys’ LG PCSA was calculated by using the high end of this range, 300 kPa (Figure 1A, black dashed line).

### 2.3. Collagen Content

A hydroxyproline colorimetric assay kit (K-555, BioVision, San Francisco, CA, USA) was used to determine the collagen content of the lateral gastrocnemius muscle from juvenile and adult domestic turkeys as well as adult wild turkeys. Hydroxyproline is an amino acid that is almost solely found in collagen and elastin in animals and can therefore be used as a direct measurement for collagen concentration within a tissue [32]. Elastin content in the muscles was not determined. Elastin in skeletal muscle is approximately 4% of the amount of collagen present [33], and is not expected to influence results from the hydroxyproline assay. Muscle samples weighing around 5 g were cut from the center of each LG, taking care not to include any tendon or aponeurosis material. Samples were homogenized in a 7 mL glass homogenizer with 100 µL dH_2_O for every 10 mg of tissue. Triplicates of 100 µL of homogenate were hydrolyzed in 100 µL of HCl (12N) for 3 h at 120 °C. Then, 10 µL of each sample (final dilution factor = 22) in duplicate were dried at 60 °C overnight in a 96-well plate. One of each duplicate was spiked with 4 µg of standard to correct for interference from endogenous compounds. Chloramine-T solution was added to each dried sample and incubated for 5 min followed by Ehrlich’s reagent, or DMAB (*p*-dimethylamino benzaldehyde), incubated at 60 °C for 90 min. The concentration of hydroxyproline was then determined by spectrophotometry at 560 nm and normalized to the mass of the sample. A standard curve was generated from known concentrations of hydroxyproline corrected from a background reading. The spiked sample readings were corrected with their corresponding non-spiked sample reading to calculate hydroxyproline µg/mg of muscle for each turkey. Collagen concentration was then calculated, assuming collagen weighs 7.5 times the measured hydroxyproline concentration [34].

### 2.4. Collagen Imaging

Fresh muscle samples taken from the mid-section of each LG, with aponeurosis intact for fiber orientation reference, were fixed in 10% formalin immediately after their removal, following previously published methods [35]. Samples were taken from two wild birds, six juvenile domestic birds and four adult domestic birds, which contained a portion of aponeurosis for context, and then subdivided into 3–5 smaller portions for further processing. Each sample was then fixed in Karnovsky’s solution overnight, which resulted in a slightly more rigid sample, before being cut into a roughly 1 mm by 5 mm slice, perpendicular to the direction of the fibers. Samples were then washed with 0.1 M sodium cacodylate buffer at pH 7.4 twice for an hour. To digest the muscle and leave collagenous structure intact, the tissue was placed in 10% aqueous sodium hydroxide for up to 7 days at room temperature and checked daily for progression. If the solution became too cloudy, it was replaced until the sample was clear of all muscle tissue. Once the muscle tissue was digested, the sample was washed in distilled water for up to 3 days.

The samples were then prepared for imaging by first washing in 1% tannic acid for 3 h, followed by another three-hour wash in distilled water. They were then soaked in 1% Osmium tetroxide overnight. After two 30 min distilled water washes, the samples were dehydrated in an ethanol series: 30%, 50%, 70%, 95%, 100%, 100% and 100% for 15 min each. The ethanol was then eliminated via critical point drying (Ladd Research Industries, Williston, VT, USA) and the samples were mounted on an aluminum stub and coated in gold with a sputter coater (Emitech K550 and Emitech K100x glow discharge unit, Quorum Technologies, East Sussex, UK). A scanning electron microscope (Hitachi S-2700, Hitachi High-Technologies, Tokyo, Japan) equipped with a lanthanum hexaboride gun was then used to view the tissue and images were collected with a Quartz PCI digital imaging system (Quartz Imaging Corporation, Vancouver, BC, Canada).

Analysis of the SEM images was performed using ImageJ [36]. The scale was recorded during image data collection for measurement reference. The endomysium interior area was used as a proxy for fiber area, as this was the space the muscle cells occupied before being digested away for SEM, and measured using the threshold tool. Step by step image analysis instructions can be found in the supplementary information. Fiber density was also estimated by counting the number of fibers in a given area of the wide view images.

Average fiber areas were found for other species to compare across a wide range of body mass (over 10,000-fold, finch to human). The average fiber cross-sectional area for the LG in the finch, pigeon, chucker, mallard, pheasant, chicken and goose were obtained from Snyder [37], covering a body mass range of 11.8 g to 5.5 kg. The fiber cross sectional area for an emu M. gastrocnemius pars medialis was measured from a figure using the same methods we used for our data [38], which may be a slight under estimate of the fiber area in the LG based on fiber type proportions reported. The average LG fiber areas for mice [39], rat [40], male humans [41], male ostriches [42] and tufted ducks [43] were also included.

### 2.5. Statistics

The relationship between PCSA and body mass between strains, as well as the relationship between force and PCSA, were compared using standard major axis regressions in SMATR using R [44]. The hydroxyproline concentrations and fiber areas for each group of turkeys were compared with Student’s *t*-test for age (juvenile domestic vs. adult domestic) and strain (adult domestic vs. adult wild) in JMP Pro 15.0 (64 bit, SAS Institute, Cary, NC, USA).

## 3. Results

### 3.1. Muscle Area and Force Scaling

Measured peak muscle force in the domestic turkey LG was compared with predicted peak force, calculated from measured PCSA and a typical value of force/PCSA for vertebrate muscle (30 N cm^−2^, [31]). The peak isometric force in the domestic turkeys increased with a slope of 33.6 N cm^−2^ (r^2^ = 0.92) (Figure 1A). This was not significantly different from the predicted slope of 30 N cm^−2^ (*p* = 0.149). The LG PCSA increased with body mass in both strains, with wild turkey PCSA scaling with body mass^0.93^ (r^2^ = 0.35) and domestic turkey PCSA scaling with body mass^0.79^ (r^2^ = 0.97). The slopes were not significantly different between strains (*p* = 0.413). Scaling in the domestic turkey was significantly greater than the expected slope of 0.67 for geometric scaling (F = 12.296, *p* = 0.003) (Figure 1B). For a given body mass, wild turkey PCSA was slightly higher, in general, as reflected by a shift in elevation of the slope (*p* = 0.001). 

### 3.2. Collagen Quantitative and Qualitative Concentration

**Figure 2:**
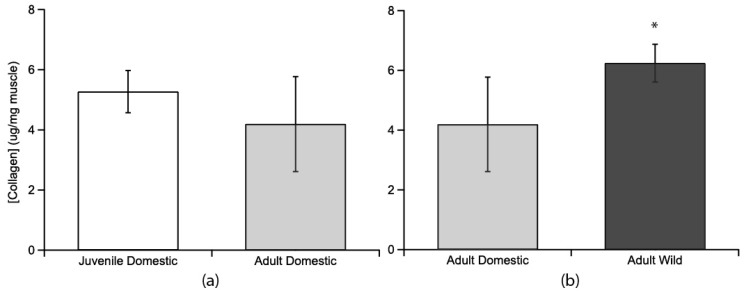
Collagen concentrations as determined from hydroxyproline concentrations for the lateral gastrocnemius muscle compared across age and strain. (**a**) Juvenile domestic collagen concentration (no fill, *n* = 6) was not significantly different from adult collagen concentration in domestic birds (light grey fill, *n* = 4). (**b**) Adult wild turkeys’ LG muscle (dark grey fill, *n* = 4) had a significantly higher collagen concentration (indicated by *) than adult domestic turkey LG muscle. Error bars are standard error of the mean (s.e.m.).

Collagen content, as measured by hydroxyproline assay, was compared between age groups and between strains (Figure 2). Juvenile domestic (5.27 ± 0.70 µg hydroxyproline/mg of wet muscle tissue; mean ± s.e.m.) and adult domestic turkeys (4.19 ± 1.58 µg/mg) were similar (*p* = 0.387, Figure 2a). Adult wild turkeys (6.23 ± 0.63 µg/mg) had a higher collagen content than adult domestic turkeys (4.19 ± 1.58 µg/mg) (*p* = 0.0275, Figure 2b).

**Figure 3:**
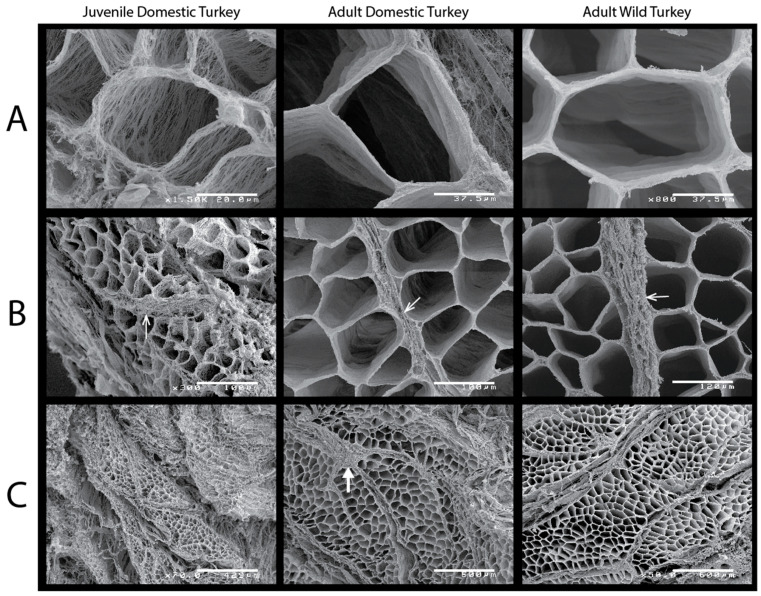
Scanning electron microscope images of collagen in turkey muscle. (**A**) Close up of endomysium. Note individual collagen fibrils are discernable in the juvenile domestic turkey (scale bar = 20 μm). The endomysium encloses similar areas in the adult domestic and wild turkey (bars = 37.5 μm), which is the value we used to estimate the cross-sectional area of muscle fibers. (**B**) Image showing perimysium (indicated by line arrows). The perimysium tended to get pulled apart in the young domestic turkeys (image scale bars are, from left to right, 100, 100 and 120 μm). (**C**) Fascicles (collections of muscle fibers bound by perimysium) can be made out in the largest scaled images (from left to right, bar = 429, 600 and 600 μm). Adult muscle fascicle sizes are very similar between strains, while the juvenile domestic birds had wispy and less neatly organized collagen overall. A white arrow indicates a fat deposit in the adult domestic turkey.

Endomysium and perimysium collagen structure were characterized with SEM images. Qualitatively, there were visible differences in the collagen distribution and amount among the three turkey groups (Figure 3). The juvenile domestic turkey muscle was very difficult to section for SEM analysis because it shreds at the fascicle level very easily. The endomysium of the young domestic turkeys was much wispier than either adult strain, with defined individual collagen fibers and gaps between them (Figure 3A). Intramuscular fat was found in both the adult wild and domestic samples within the perimysium and in larger deposits (indicated by the white arrow in Figure 3C). 

### 3.3. Fiber Area

**Figure 4:**
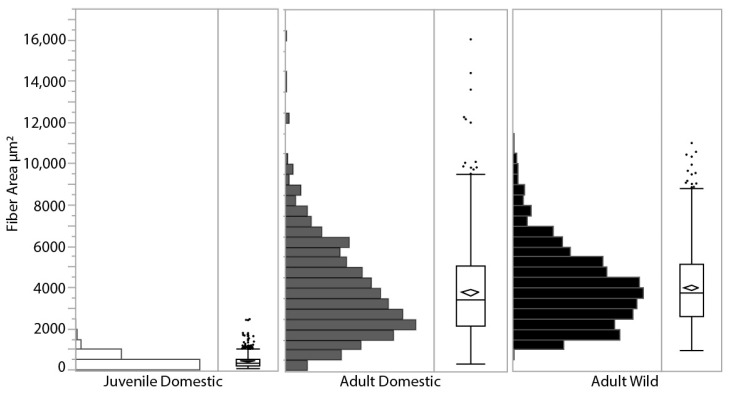
Muscle fiber cross-sectional area distribution for each group of turkeys, measured as the area enclosed by the endomysium from SEM images. The diamond within the box plot indicates the mean for each group, the box represents the 25% quartile, median and 75% quartile, with markers indicating outliers. Juvenile domestic turkeys (open bars, *n* = 6 turkeys, 1297 fibers mean = 428 ± 284 μm^2^) were significantly smaller than the adult domestic turkeys (grey bars, *n* = 4 turkeys, 872 fibers mean = 3802 ± 2223 μm^2^), displaying the hypertrophy that occurs during growth as the fiber cross-sectional area increases. Mean adult wild turkey fiber area (black bars, *n* = 2 turkeys, 848 fibers, mean = 4014 ± 1831 μm^2^) was significantly greater than adult domestic turkeys.

LG muscle fiber cross-sectional area was measured from SEM images in all three turkey groups and showed a range of size distributions (Figure 4). Adult wild turkeys had a larger mean fiber area (mean = 4014 ± 1831 μm^2^, *n* = 2 birds totaling 848 fiber areas measured), which was significantly greater than adult domestic turkeys (mean = 3802 ± 2223 μm^2^, *n* = 4 birds totaling 872 fiber areas measured) (*p* = 0.013). Average adult domestic turkeys’ fiber areas were over 11 times larger than juvenile domestic turkeys’ (mean = 428 ± 284 μm^2^, *n* = 6 birds totaling 1297 fiber areas) (*p* < 0.0001). Based on fiber density estimates reported during the fiber area measurements of 1160 fibers mm^−2^ in the juvenile turkeys and 200 fibers mm^−2^ in the adult domestic turkeys, and using the PCSA of the whole muscle (Figure 1A), the number of muscle fibers stays roughly constant in the LG muscle during growth, at approximately 500,000 fibers.

**Figure 5:**
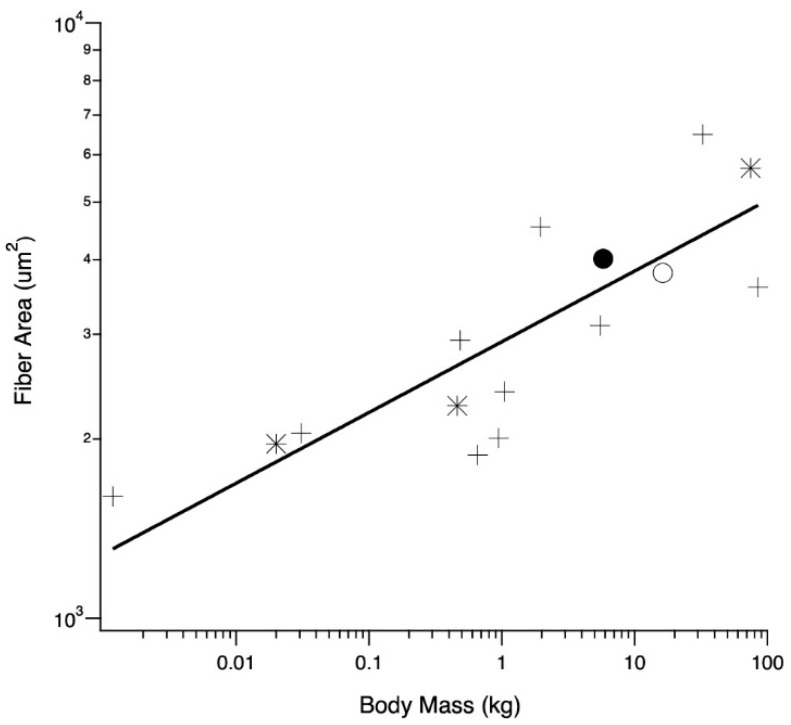
Relationship between body mass and muscle fiber cross-sectional area for birds (crosses), mammals (asterisks), adult wild (closed circle) and adult domestic (open circle) turkeys. All but one mean fiber area are taken from the LG; the emu (body mass of 32.5 kg) is taken from the M. gastrocnemius pars medialis [38]. Other values are mice, [39], rat [40], male humans [41], male ostriches [42] and tufted ducks [43]. Coefficient and exponent of the allometric equation y = aM^b^, where M is body mass, a = 3.46 and b = 0.11 (r^2^ = 0.67).

The relationship between average LG (and one medial gastrocnemius) fiber cross-sectional areas and body mass was examined across a wide size range of species, providing context for averages observed in adult wild and domestic turkeys (Figure 5). Across all species, fiber area scaled with body mass^0.11^ (r^2^ = 0.67), and the regression was significant (*p* = 0.0002).

## 4. Discussion

Based on the selective regime that domestic turkeys have undergone, and known locomotor issues, we expected to find significant changes to the LG muscle’s structure and function. We found the differences in the LG muscle to be minimal between strains. The domestic turkey LG could produce the expected amount of force per area and the muscle fiber cross-sectional areas were not larger than the wild turkeys, as one might predict based on selection for increased meat production. We did find differences in the connective tissue of the LG extracellular matrix between wild and domestic turkeys, and these may have some functional consequences. However, even where we found similarities in the LG structure and function in these large turkeys, there are implications for locomotion and movement, which we address below. 

### 4.1. Muscle Function

The domestic turkey reaches a total body mass 3× that of a wild turkey, predominantly a result of increased muscle masses due to artificial selection [2]. However, selection for increased muscle mass and possible relaxed selection on muscle function has not altered the ability of the lateral gastrocnemius muscle to produce force in the domestic turkey. We expected to find reduced force output based on reports of leg weakness and locomotor issues in domestic turkeys [6,19,45,46]. Remarkably, the LG muscle can still produce the expected force per PCSA, i.e., peak stress (Figure 1B). In fact, the domestic turkey LG muscle is on the high end for skeletal muscle [31], averaging 29.2 ± 5.2 N cm^−2^ The LG is most active during the stance phase of locomotion [28,47], and so the sustained force production of the lateral gastrocnemius muscle may be influenced by the continued importance of this muscle for terrestrial locomotion and the act of standing in the domestic turkeys.

However, simple scaling issues still affect the domestic turkey. Despite the maintained peak muscle force performance, domestic turkey LG muscles are still relatively weaker than wild turkeys due to the large body masses domestic turkeys attain [2]. The PCSA scaled with body mass faster than the hypothesized geometric relationship (0.67) with a slope of 0.79 (Figure 1A), but not fast enough to keep up with the force production needs due to increased body mass (which would be indicated by a slope = 1). The observed scaling relationship would indicate that a domestic turkey three times the body mass of its wild counterpart would generate only 2.4× the LG muscle force. This finding is similar to those of studies comparing architectural properties of modern broiler chickens with those of Giant junglefowl, which found a decline in pelvic limb muscle mass, likely compromising locomotor capabilities [48]. In addition, the domestic turkey has experienced a shift in its center of mass [11], likely altering the effective mechanical advantage of many hind limb muscles. This could partially explain the locomotor differences observed between strains, as domestic turkeys are limited to slow speeds and relatively low vertical ground reaction forces [11].

### 4.2. Muscle Collagen Content

Consumer preference for poultry meat is driven by appearance and texture [49]. Meat tenderness, a mechanical measure, is a major component contributing to these qualities [50]. Meat quality changes, including tenderness, have been associated with intensive genetic selection in the turkey industry [4,18,51]. Many genes associated with muscle qualities have been identified as being differentially expressed in selected turkey lines, including genes regulating extracellular matrix (ECM) within the muscle, the major contributor to meat tenderness [52]. Meat tenderness is correlated with the amount of connective tissue predominantly at the fascicle level within the perimysium, which is made up mostly of collagen [20,53]. We found that the adult domestic turkey LG muscles had significantly less collagen content than the adult wild turkeys. This result is similar to other poultry studies that found that the wild counterpart to chickens, Red junglefowl, had muscles with higher collagen content than commercial broilers [54,55]. Our results are consistent with the idea that selection for increased tenderness has led to a reduction in connective tissue in the domestic turkey compared to the wild turkey.

The collagen concentration is not significantly different between juvenile domestic and adult domestic turkeys, indicating that the collagen content in a given volume of muscle is similar (Figure 2). This is perhaps surprising as we expect muscle to increase both perimysium and endomysium thickness with age, as the muscle stiffens during the aging process [14]. The similarity in collagen content between adults and juveniles can be explained by the counteracting effects of change in fiber area and change in thickness of epi/perimysial layers. Given the difference in fiber area (Figure 4), about 10 juvenile fibers can fit within the area of an adult domestic muscle fiber. Assuming equal endomysial thickness, smaller fibers should be associated with more collagen simply because there is a greater total perimeter of cell boundaries. Based on the circumferences of those fibers, the juvenile domestic turkey would have about 708 μm of endomysium within that area (10 fibers) and the adult domestic turkey would have just 225 μm of endomysium (1 fiber). Since the collagen amounts are the same in a given volume, we would then expect the juvenile domestic turkey endomysium collagen to be over 3 times less dense, or thick. Indeed, the collagen density differences are reflected in the close-up SEM images where the endomysium of the juvenile domestic turkeys is not opaque, and looks more like a web of collagen rather than a wall (Figure 3A). Of course, the density is qualitative in this study, and there are likely differences in the perimysium as well. The hydroxyproline assay we used to determine collagen concentration does not account for chemical differences in collagen types and factors such as collagen cross-linking, which also affect the mechanical features of the ECM [14,56,57].

Intramuscular fat deposits can also affect the structure and mechanical properties of the muscle. We saw evidence of intramuscular fat throughout the adult domestic turkey muscle samples (Figure 3C), and a few within the wild turkey samples, as well. Intramuscular fat can form within the perimysium, resulting in marbling, also called white striping in poultry [16,58]. Intramuscular fat deposits cause remodeling and disorganization of the ECM, resulting in more tender meat [59]. These structural changes within the ECM could affect muscle function in the animal, similar to mechanical changes seen in the muscle of aged rats [13].

### 4.3. Effects of Selection on Fiber Size

Muscles can dramatically increase in size during growth and with activity, with whole muscle size being altered by an increase in the number of fibers or an increase in individual fiber size. Existing studies show increases in fiber size occur with activity and growth [15,60], but it is less clear whether there is an increase in fiber number or size with body mass across species or even strains. Past studies have shown that the number of muscle fibers is determined during embryonic development [61], and muscle fiber hypertrophy accounts for the increase in size during ontogeny [62,63]. In the turkey pectoralis, for example, fiber cross-sectional area increases by 36 times during the first 15 weeks of growth [60]. Our data from the juvenile and adult domestic turkeys also support fiber hypertrophy with growth (Figure 4), with fiber cross-sectional area increasing by about 11 times from approximately 8 to 18 weeks in age. Between poultry lines, fiber size in chickens has been shown to increase in the faster growing lines and with muscle weight [4,64], and fast-growth farm animals have more muscle fibers than slow growth lines [65]. 

Due to selection for larger muscle mass, we expected the domestic turkey fiber areas to be larger than the wild birds and for them to have more fibers within the muscle overall. Surprisingly, wild turkey muscle fibers turned out to have a greater average cross-sectional area than domestic turkey muscle fibers in this study (Figure 4). Since the entire LG muscle of a domestic turkey has a larger PCSA than a wild turkey, meaning the muscle is overall larger in cross-section due to selection for increased muscle size for meat, they must contain many more fibers than the wild turkeys. As turkeys are one of the most recently domesticated animals and diverged only recently in evolutionary history, around 700 C.E. [66], we can assume that the original wild type had similar muscles to today’s wild turkey. Thus, artificial selection for increased muscle mass has led to an increase in the number of fibers (i.e., hyperplasia) rather than an increase in fiber size (i.e., hypertrophy) in the LG of this strain of turkeys. In the future, it would be beneficial to examine the fiber type composition in each strain, given this fiber cross-sectional area divergence. 

Domestic turkey LG muscles can be more than twice the size of wild turkey LG muscles and yet the domestic turkeys have slightly smaller fiber cross-sectional areas. One potential explanation for this surprising pattern is that the turkeys are running into some sort of absolute limit of muscle fiber area. To examine this idea, we gathered data on fiber size for a range of animals from the literature (Figure 5). The data show a positive correlation between body mass and fiber size. There also does not appear to be evidence for any sort of absolute limit on fiber size, as it increases for body weights up to 100 kg, well above the size of our turkeys. The scaling of total muscle cross-sectional area with body mass has been derived as M^0.80^ [31], and our LG PCSA results support this (Figure 1B). It seems that across animals of different body mass, an increase in fiber size does account for a certain proportion of the increase in muscle mass associated with an increase in body mass. However, this is not the case for the increase in muscle mass of the domestic turkey. Increasing the overall muscle mass with fiber number alone could help preserve function in the LG. Functional consequences for increasing fiber size include decreased connective tissue spacing, altering diffusion distances or inadequacies in capillary supply, as seen in the pectoralis muscle [17,67,68,69]. 

## 5. Conclusions

Selection for increased body mass and muscle mass has resulted in structural changes within the lateral gastrocnemius muscle of the domestic turkey. An increase in the number of muscle fibers is responsible for the increase in the LG cross-sectional area with body mass between the wild and domestic turkey strains. Collagen concentration was significantly decreased in the domestic turkey, which may reflect selection for tenderness. Despite these changes, the muscle was still able to produce the expected amount of force per physiological cross-sectional area. However, because the domestic turkeys reach much higher body masses, scaling has still resulted in domestic turkeys producing less force per unit body mass than wild turkeys. Force production is just one measure of muscle performance; the structural changes we observed in the intramuscular connective tissue of the domestic turkey muscle could have other functional implications for these large birds. 

## Data Availability

The data presented in this study are openly available in FigShare at: 10.6084/m9.figshare.14806839, 10.6084/m9.figshare.14806707, 10.6084/m9.figshare.14806731.

## Supplementary Materials

The following are available online at https://www.mdpi.com/article/10.3390/ani11071850/s1. Methods S1: SEM analysis in ImageJ. Table S1: Individual turkey information from SEM imaging, fiber area analysis and hydroxyproline assay. All data are available upon reasonable request.
