# Supplementary material for: Gastrocnemius Muscle Structural and Functional Changes Associated with Domestication in the Turkey
Source: Animals (Basel). 2021 Jun 22;11(7):1850. doi: 10.3390/ani11071850 (PMC8300382; doi:10.3390/ani11071850)
Supplement: Supplementary file 1 [file animals-11-01850-s001.zip › animals-1249961-supplementary.pdf]

Supplementary Information

Muscle structural and functional changes associated with domestication in the turkey

Methods S1. SEM analysis in ImageJ is described below. The scale was recorded during SEM image data collection for measurement reference. The endomysium interior area was used a proxy for fiber area, as this was the space the muscle cells occupied before being digested away for SEM, and was measured using the threshold tool as described here.

1. Acquire SEM image and save under **.TIF** format
2. Download and Open ImageJ
3. **File → Open → Select Photo**
    a. Download and save the photo with preferred title
4. To set scale:
    a) Select the **straight/segmented/freehand line** tool on the ImageJ Toolbar.

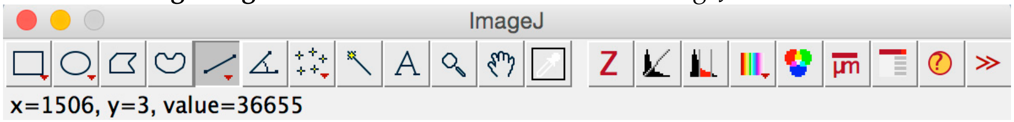

    b) Trace the image's provided scale bar. Hold shift while drawing the line to ensure a horizontal measurement and maintain consistent method of tracing the reference line across images
    c) Analyze → Set Scale
    d) Change the values for **known distance** and **unit of length** according to determine pixel to given unit ratio (ie. 600, um).
    e) Record the ratio of pixels to provided units for reference.
    f) Select global if pixel to unit ratio is constant across image series (if not constant, repeat step before analyzing each image of the series).
    g) Hit "OK"
5. **Image → Duplicate**
    a) Save and rename duplicated image accordingly
    b) This step provides a photo to reference and compare to once image edits are underway. Any opened image file will suffice.
6. To analyze image through manual method:
    a. Select **Freehand Selections**

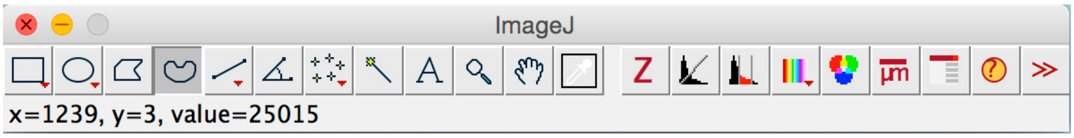

    b. Trace the perimeter of a muscle fiber
    c. Analyze → Measure
    d. Analyze → Label
    e. Repeat steps 6b-6d for **each** traced muscle fiber. The areas will be saved in the **Results** window that opens once **Measure** is selected; however, in order to save the trace and corresponding numerical label, one must select **Label** immediately after measuring each muscle fiber area.
    f. Once finished tracing, measuring, and labeling muscle fiber areas, save the **Results** with appropriate title, screenshot labeled image for reference, and transfer data to record sheet.

7. To analyze image through threshold method:

a) Image → Adjust → Threshold

Adjust the two bars in order to select the range of grey shades to include in analysis.

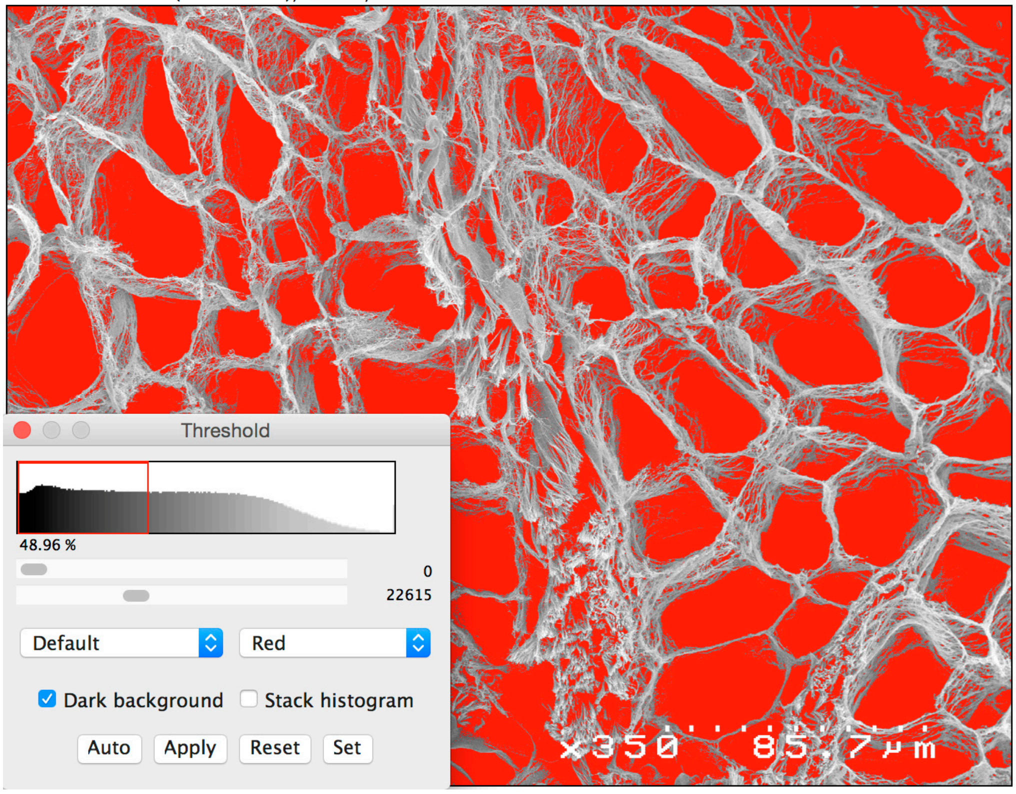

1. Top bar determines lower limit.
2. Bottom bar determines upper limit.
3. Specifically, for fiber area measurement, set the top bar to "0" and adjusted the bottom bar to include as much of the dark space of the desired muscle fibers as possible without selecting (and thus removing) fiber borders.

i. Select **Dark Background**

ii. Hit **Apply**

iii. To remove outliers and scattered particles:

i. Process → Noise → Despeckle

ii. Process → Noise → Remove Outliers

1. Determine the radius (in pixels as that is the given unit for this selection) of the minimal area to be included in the analysis and input value.

2. Threshold selection relates to the deviation from the median area outside of which will be excluded.

3. Select Dark (in order to exclude dark particles that are smaller than the muscle fibers to be measured).

4. Select Preview and play with values until image represents selection as close to preferred selection as possible.

a. To edit out remaining undesired areas:

Select **Polygon Selection, Freehand Selections, Oval/Rectangular Selections**, etc. on the ImageJ Toolbar (whatever best suits desired editing) and outline areas to be removed.

. **Edit → Fill** (this will "white out" selected area so that it will not be included in the analysis)

i. Repeat as necessary (can select one area at a time)

ii. To deselect: **Edit → Selection → Select None**

b. Analyze → Analyze Particles

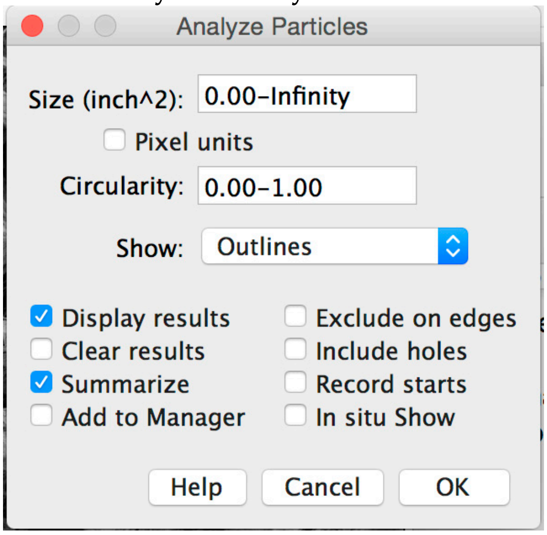

Size:

1. Ensure appropriate units indicated (so um^2 rather than inch^2).

2. List minimum area (in appropriate unit) to be included in analysis.

i. Show: Select **Outlines** from drop-down menu.

ii. Check **Display Results** and **Summarize**

iii. Circularity refers to the shape of particles to be included. A selection of "0.00-1.00" indicates that all shapes from a line to a perfect circle will be included, with "0.00" indicating a line and "1.00" indicating a perfect circle.

iv. Hit **OK** when finished with selections.

v. Manually inspect results to see what modifications to size inclusion/image edits are necessary to provide the desired results. Go back and change as necessary.

Save **Results** with desired name and screenshot the outlined image for reference later when determining which fiber corresponds to each measured area. Screenshot image edits and input data into record sheets.

**The threshold method and manual method may not give consistent results in that the threshold method normally underestimates muscle fiber area. The threshold method is best utilized when there are too many fibers to manually trace or when magnification is too low to properly visualize (human limitation) to measure the fibers. Otherwise, the manual method is optimal and allows the user to be more selective with which fibers to include in the analysis.

Table S1. Individual turkey information for SEM imaging, fiber area analysis, and hydroxyproline assay

**Group**	**Turkey ID**	**Bird Mass (kg)**	**LG Mass (g)**	**Age (weeks)**	**Mean Fiber area (um^2)**	**Standard Error**
Wild	WM1	3.9	21.18	20	5580	196
	WM2	7.7	59.56	>52	3899	53
	WF1*	4.9	22.29	>52		
	WF2*	4.2	21.62	>52		
Juvenile domestic	YD1	1.8	8.61	8	412	158
	YD2	2.1	8.16	9	280	225
	YD3	2.3	13.17	10	402	67
	YD4	3.0	15.48	10	625	128
	YD5	3.3	13.71	10	425	76
	YD6	2.8	13.55	11	391	124
Adult Domestic	OD1	16.8	84.24	30	2334	130
	OD2	16.4	95.17	30	4271	78
	OD3	17.0	82.79	30	3731	107
	OD4	15.0	71.29	30	4004	114

*Only hydroxyproline assay
